# The cullin Rtt101 promotes ubiquitin-dependent DNA−protein crosslink repair across the cell cycle

**DOI:** 10.1093/nar/gkae658

**Published:** 2024-07-30

**Authors:** Audrey Noireterre, Julien Soudet, Ivona Bagdiul, Françoise Stutz

**Affiliations:** Department of Molecular and Cellular Biology, University of Geneva, 1211 Geneva 4, Switzerland; Department of Molecular and Cellular Biology, University of Geneva, 1211 Geneva 4, Switzerland; Department of Molecular and Cellular Biology, University of Geneva, 1211 Geneva 4, Switzerland; Department of Molecular and Cellular Biology, University of Geneva, 1211 Geneva 4, Switzerland

## Abstract

DNA−protein crosslinks (DPCs) challenge faithful DNA replication and smooth passage of genomic information. Our study unveils the cullin E3 ubiquitin ligase Rtt101 as a DPC repair factor. Genetic analyses demonstrate that Rtt101 is essential for resistance to a wide range of DPC types including topoisomerase 1 crosslinks, in the same pathway as the ubiquitin-dependent aspartic protease Ddi1. Using an *in vivo* inducible Top1-mimicking DPC system, we reveal the significant impact of Rtt101 ubiquitination on DPC removal across different cell cycle phases. High-throughput methods coupled with next-generation sequencing specifically highlight the association of Rtt101 with replisomes as well as colocalization with DPCs. Our findings establish Rtt101 as a main contributor to DPC repair throughout the yeast cell cycle.

## Graphical Abstract

**Graphical Abstract:**
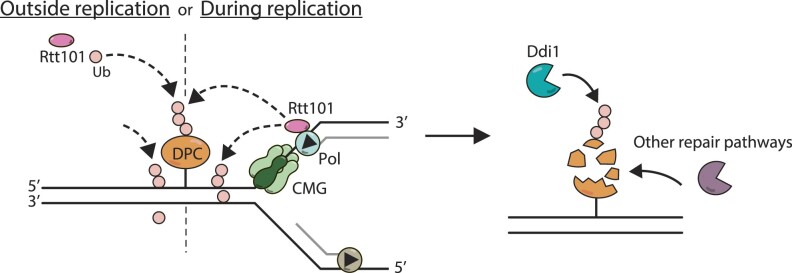

## Introduction

Cells have evolved a repertoire of repair pathways to rapidly counteract DNA damage and ensure genome integrity. Proteases are able to accurately catalyze the hydrolysis of peptide bonds in protein substrates (1), a feature that often places them at the core of these specialized mechanisms. Such potent enzymes, however, must be tightly regulated to avoid unwanted, excessive or unrestrained proteolytic activity that can eventually cause a variety of human pathologies (2).

Proteins covalently attached to the DNA form stable structures known as DNA–protein crosslinks (DPCs) which pose a threat to DNA integrity by interfering with fundamental processes such as transcription or replication (3,4). DPCs arise from exposure to exogenous agents (chemotherapeutics, UV, IR, etc.) (5,6) or from unspecific crosslinking induced by endogenous metabolites (7,8,9). The processing of these bulky lesions in yeast and higher organisms relies on a growing list of DPC proteases [reviewed in (10,11)]. Until now, the involvement of the following proteases in DPC repair has been demonstrated: Wss1/SPRTN (3,12,13,14,15,16,17), 26S proteasome (18,19,20,21,22), Ddi1 (23,24), FAM111A (25) and ACRC/GCNA (26,27,28). Additionally, canonical repair pathways such as nucleotide excision repair (NER) and homologous recombination (HR) are also taking part in the resistance against DPCs, highlighting the diversity of repair options available to cells, (29), that need to be finely tuned to select the most appropriate repair outcome.

Post-translational modifications (PTMs), including ubiquitination, sumoylation, acetylation and phosphorylation are regulating some facets of DPC processes (22,26,30,31,32,33,34,35). In particular, ubiquitin emerged as a pivotal player in overcoming DPC lesions, but the complexity of the pathways involved is still not fully elucidated. In higher eukaryotes, the RING-type E3 ubiquitin ligase TRAIP is a canonical replisome component targeting DPCs for ubiquitination when encountered during the replication (36). This event triggers the bypass of DPC lesions by the replicative CMG (CDC45, MCM2-7, GINS) helicase (37). While TRAIP-dependent ubiquitination is not required for SPRTN action on DPCs, it is stimulating the activity of the proteasome (22). RFWD3 is another ubiquitin ligase triggering additional DPC ubiquitylation (38). In the absence of TRAIP, CMG bypass of DPC lesions is delayed, however the adduct is still ubiquitinated, likely by RFWD3 (38,39). In yeast, the SUMO-dependent ubiquitin ligase (STUbL) Slx5-Slx8 (hRNF4) participates in the Wss1-dependent Top1cc processing (32). The human STUbL RNF4 is promoting replication-independent proteasomal degradation of DPCs (30,31), adding another layer of complexity to the role of ubiquitin in DPC repair. However, there is currently no replication-associated E3 ubiquitin ligase linked to DPC repair in yeast.

Through genetic screening, genetic interactions, molecular analyses, and high-throughput techniques, our study identifies the cullin RING E3-ubiquitin ligase Rtt101 (also known as Cul8) as a new candidate regulating DPC repair in the yeast *Saccharomyces cerevisiae*. The mammalian homolog of Rtt101 (CUL4B) was notably shown to counteract DPCs that arise following chemotherapeutic treatment in mammalian cells (40). Although yeast Rtt101 was previously implicated in general DNA repair mechanisms and in ensuring the progression of the DNA replication machinery through damage sites (41,42,43,44,45), its direct association with DPC repair has not been established.

We found that functional Rtt101-E3 ligase complexes are required to counteract DPCs, and the DPC protease Ddi1 is part of a pathway dependent on Rtt101. Using an *in vivo* inducible site-specific DPC model, we provide evidence that the ubiquitination activity of Rtt101 locally influences DPC removal from the DNA. We show that Rtt101 is associated with replisomes, although its activity is also remarkably required outside of S-phase. Collectively, our results indicate that Rtt101 most likely functions as a regulator that facilitates DNA–protein crosslink repair at different stages of the cell cycle in budding yeast.

## Materials and methods

### Resource availability

Further information and requests for resources and reagents should be directed to the lead contact, Françoise Stutz (francoise.stutz@unige.ch).

### Antibodies, reagents, kits, chemicals and instruments

The following antibodies were used for western blot: Mouse anti-polyhistidine (monoclonal, HIS-1; Sigma-Aldrich, cat# H1029; RRID:AB_260015; dilution 1:2000); Mouse anti-PGK1 (monoclonal, clone 22C5D8; Abcam, cat# ab113687; RRID: AB_10861977; dilution 1:3000); Rabbit anti-Histone H3 (polyclonal; Invitrogen; cat# Pa5-16183; RRID: AB_10985434; dilution 1:2000); Mouse anti-HA (monoclonal, clone 16B12; Biolegend; cat# 901502; RRID: AB_2565006; dilution 1:2000); Mouse anti-MYC tag (monoclonal, clone 9E10; Abcam; cat# ab32; RRID:AB_303599; dilution 1:5000); Fluorescent secondary Goat IRDye 800CW anti-mouse (LI-COR; cat# 926-32210; RRID: AB_621842; dilution 1:4000; Secondary Goat anti-Mouse-HRP (DAKO; cat# P0447; RRID: AB_2617137; dilution 1:5000); Secondary Goat anti-Rabbit-HRP (DAKO; cat# P0448; RRID:AB_2617138; dilution 1:5000).

The following antibodies were used for ChIP: Mouse anti-HA (monoclonal, clone 16B12; Biolegend; cat# 901502; RRID: AB_2565006; ChIP 1 μg/1 mg protein); Mouse anti-ubiquitin (monoclonal, clone FK2; Calbiochem; cat# ST1200; RRID: AB_2043482; ChIP 1 μg/1 mg protein); Mouse anti-FLAG (monoclonal, clone M2; Sigma-Aldrich; cat# F3165; RRID:AB_259529).

The detailed list of chemicals, critical commercial assays, software and instruments can be found in Supplementary Table S1.

### Yeast strains and growth conditions

Strains used in this study (listed in Supplementary Table S1) are standards *Saccharomyces cerevisiae* W303 [*leu2-3 112 trp1-1 can1-100 ura3-1 ade2-1 his3-11,15 rad5-535*] or BY4741/4742 [*his3Δ1 leu2Δ0 lys2Δ0 met15Δ0 ura3Δ0*]. If not mentioned otherwise, yeast cells were grown at 30°C in YEP- (1% yeast extract, 2% peptone) or SC- (1.7 g/l yeast nitrogen base; 5 g/l ammonium sulfate; 0.87 g/l dropout mix) liquid media or grown on plates supplemented with 20 g/l agar. As a source of sugar, 2% glucose, 2% raffinose or 2–3% galactose was added. Selection for dominant markers was performed on YEPD-based medium (YEP–2% glucose) supplemented with 200 μg/ml G418, 200 μg/ml cloNAT or 50 μg/ml Hygromycin B.

### *E. coli* strains and growth conditions

DH5α *Escherichia coli* bacterial strains were grown at 37°C in LB medium or on LB 2% agar plates supplemented with 50 μg/ml of ampicillin for plasmid selection.

### Construction of recombinant DNA

Recombinant plasmid DNA was constructed using the NEB Builder HiFi DNA assembly cloning kit (NEB, cat# E5520) following the manufacturer's recommendations. The newly generated plasmid DNA was purified using the PureYield Plasmid Miniprep System (Promega, cat# A1222), and validated by Sanger sequencing. The list of recombinant plasmid DNA used in the study can be found in Supplementary Table S1.

### Yeast transformation

Cells were grown until reaching mid-log phase at 30°C, washed with LiTe buffer (100 mM LiAc; 10 mM Tris pH 7.5; 1 mM EDTA), and then combined with 100 μg/ml salmon sperm ssDNA, 37.28% PEG4000, and either PCR fragment or plasmid DNA to transform. The mixture was incubated at 30°C for at least 1 h. Subsequently, DMSO was added to a final concentration of 6%, followed by a heat-sock at 42°C for 10 min. Cells were then plated onto the appropriate selective medium.

### Colony PCR

To validate genomic mutations (deletions, tagging) and bacterial constructs, single colonies of yeast and bacteria were directly resuspended in 10 μl of sterile water containing oligonucleotides at a concentration of 0.5 μM, followed by the addition of 10 μl of 2× Phire Green Hot Start II PCR Master Mix (Thermo Scientific, cat# F126L). Tagging was additionally verified by immunoblotting.

### Genetic crosses and tetrad dissection

Equivalent amounts of *MATa* and *MATalpha* haploid strains were mixed in 50 μl of water and 20 μl were transferred onto YEPD medium overnight to promote diploid formation, which were then selected based on auxotrophy or antibiotic resistance. The resulting diploids were then patched onto sporulation medium (20 g/l potassium acetate; 2.2 g/l yeast extract; 0.5 g/l glucose; 0.87 g/l dropout mix; 20 g/l agar; pH 7) and incubated for 4 days at 30°C.

Sporulated strains were treated with 0.5 mg/ml Zymolyase (Amsbio, cat# 120491-1) for 5 min at room temperature, before tetrad dissection using a micromanipulator. Haploid spores were incubated at 30°C for 3 days, and then replica-plated onto selective media to assess individual genotypes.

### Spot assays

Overnight yeast cultures were diluted in the morning in 5 ml of fresh medium, and grown under continuous agitation at 30°C until they reached the exponential growth phase. One ml of each strain was collected by centrifugation at 17 949 × g for 1 min and resuspended in sterile water to an OD_600_ = 3. A sterile 96-well plate was used for the preparation of six 10-fold serial dilutions. The initial OD_600_ of these dilutions was set at 1.5. Four microliters of each dilution were spotted on agar plates supplemented with the indicated concentrations of auxin, camptothecin, etoposide, hydroxyurea, formaldehyde. Plates were imaged after at least 48 h of incubation at 30°C.

### Analysis of Top1 levels following camptothecin treatment

Overnight cultures were diluted to OD_600_ = 0.2. When cells reached OD_600_ = 0.8, camptothecin was added to a final concentration of 5 μg/ml along with 100 μg/ml cycloheximide to prevent protein synthesis. Cultures collected at different time points were subjected to TCA protein extraction and immunoblotted with anti-MYC (homemade) for analysis of Top1-13MYC levels or anti-PGK1 (Abcam, cat# ab113687 clone 22C5D8) antibodies. Fluorescent secondary antibodies (LI-COR, cat# 926-32210) were used for quantification analyses.

### Protein extraction by TCA, SDS-PAGE and western blot

One ml of yeast cultures was centrifuged 1 min at 17 949 × g and pellets were resuspended in 150 μl of 20% Trichloroacetic acid (TCA). A volume equivalent to 100 μl of glass beads was added, and samples were vortexed at room temperature for 10 minutes. Then, 500 μl of 5 % TCA was added. Tubes were mixed by gentle agitation and centrifuged for 1 min at 17 949 × g. Beads and supernatant were carefully removed, and the remaining protein pellets were resuspended in 50 μl water, 50 μl 4× Bolt LDS Sample Buffer (Invitrogen, cat# B0007), 50 μl 1 M Tris–HCl pH 9.5. The samples were incubated for 10 min at 65°C and then vortexed for 5 min. Finally, a final heating step of 5 min at 75°C was performed before loading onto SDS-PAGE gel (with a concentration ranging from 6 % to 13 %, depending on the proteins) for analysis.

**Figure 1.**
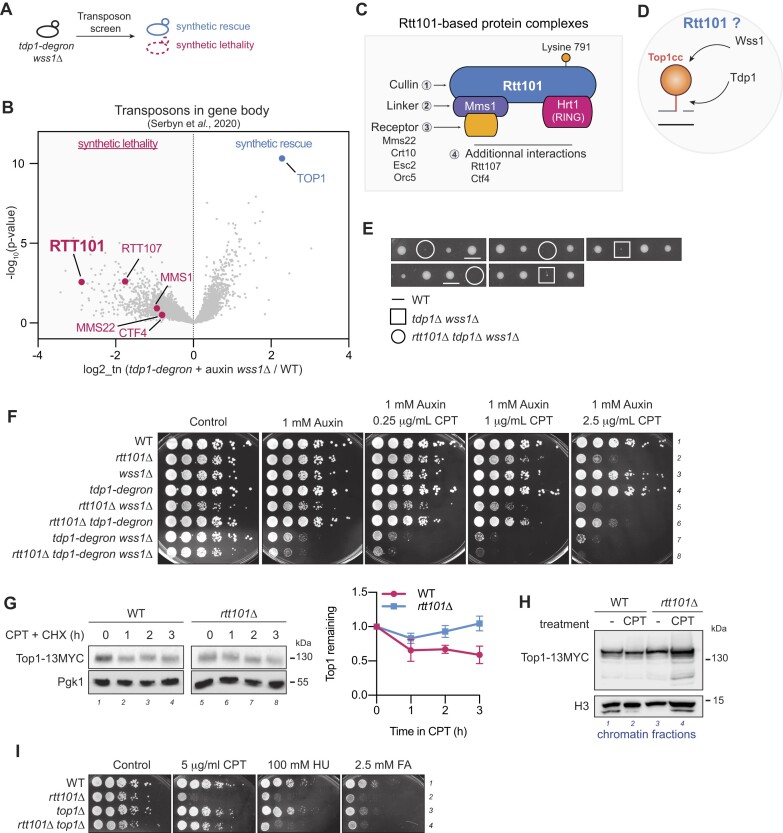
Rtt101-based E3 ubiquitin ligase participates in resistance to Top1-DNA adducts. (**A**) Unbiased yeast genetic transposon screen. Random transpositions were induced into a *tdp1-degron wss1Δ* yeast strain to generate a SATAY library, in order to identify synthetic lethal or rescue genetic interactions. (**B**) Components of Rtt101-based complexes are negatively affected by loss of Wss1 and Tdp1. Transposition events sequenced in *tdp1-degron*+ auxin *wss1Δ* compared to a pool of six unrelated libraries [results are from the SATAY transposon screen performed in (24)]. Fold-change of reads per gene (log_2_, x-axis) and corresponding *P*-values (–log_10_, y-axis) are plotted. Components of Rtt101-ubiquitin ligases presented in (C) are labelled in pink. (**C**) Schematic of Rtt101-based ubiquitin ligase complexes. The cullin Rtt101 (1; blue) is the core component of ubiquitin ligase complexes. It always carries the RING-box protein Hrt1 (pink) to mediate ubiquitination of target proteins. The linker protein Mms1 (2; purple) mediates interaction with additional receptor proteins (3; orange) for interaction with a substrate of the ubiquitin ligase complex. Finally, additional interactions (4) can occur to confine substrate specificity. (**D**) How does Rtt101 influence removal of Top1ccs, which are cleared by Wss1 and Tdp1? (**E**) Loss of *RTT101* affects growth of *tdp1Δ wss1Δ*. Tetrad analysis of *rtt101Δ* combined with *tdp1Δ wss1Δ*. Representative spores of indicated mutants are highlighted. Tetrads originate from the same YEPD plate. (**F**) Transposon screen validation of *RTT101*. Cells were grown in YEPD and spotted on a medium supplemented with 1 mM auxin to deplete Tdp1 and indicated concentrations of camptothecin (CPT). Plates were incubated for 2 days at 30°C. (**G**) CPT-induced Top1 degradation depends on Rtt101. Top1-13MYC levels were assessed at the indicated time points in exponentially growing cells in the presence of 5 μg/ml CPT and 100 μg/ml of cycloheximide (CHX) to stop protein synthesis. Top1-13MYC and Pgk1 (loading control) levels in total cell extracts were probed by immunoblotting (left panel) and quantified using fluorescent antibodies (right panel). Images show immunoblotting using X-ray films for chemiluminescence. Relative Top1-13MYC to Pgk1 levels were set to 1 in the respective non-treated samples. Graph (right panel) shows values as mean (SD) of three independent biological replicates. (**H**) Top1 accumulates on chromatin in the absence of Rtt101. Chromatin fractions were isolated from permeable *12geneΔ0HSR* cells treated for 3 h with 1.5 μg/ml CPT. Top1-13MYC protein levels on chromatin were compared by immunoblotting and revealed by chemiluminescence. H3 is used to monitor fractionation efficiency and as a loading control. (**I**) Top1 crosslinks are not the only cause of cell sensitivity to genotoxins in the absence of Rtt101. *TOP1* deletion specifically rescues CPT sensitivity of *rtt101Δ* mutant, but does not improve hydroxyurea (HU) and formaldehyde (FA) resistance. Cells were grown in liquid YEPD and plated on 5 μg/ml CPT, 100 mM HU and 2.5 mM FA. Plates were incubated for 2 days at 30°C.

Following SDS-PAGE resolution, the proteins were transferred onto a nitrocellulose membrane (GVS, cat# 1215458) using a semi-dry transfer. The membrane was blocked for 30 min with TBST (150 mM NaCl; 20 mM Tris–HCl pH 7.4; 0.05% Tween) + 5% milk. Primary antibodies were diluted to their working concentration in TBST + 5% milk and incubated overnight at 4°C. The next day, the membrane was washed four times for 5 min with 10 ml of fresh TBST, and HRP-coupled or fluorescent secondary antibodies were applied for 1 h at room temperature. For the complete list of antibodies, refer to the List of antibodies and reagents in Supplementary Table S1. The antibodies are indicated in each figure and figure legend. Fluorescence images were captured using the Li-COR Odyssey Imaging System, and chemiluminescence was detected with the Amersham ImageQuant 800 system or on X-ray films (Top1-13Myc degradation representative figure, Figure 1G).

### Isolation of chromatin

Chromatin fractions were prepared following the protocol of (46) with minor modifications. Fifty OD of yeast cells were harvested by centrifuging cultures for 3 min at 1650 × g followed by a single wash with cold 1× phosphate saline buffer (PBS). Harvested cells were frozen in liquid nitrogen. All centrifugations were performed at 4°C unless specified otherwise.

Pellets were resuspended in 1 ml of pre-spheroblasting buffer (100 mM PIPES/KOH, pH 9.4; 10 mM DTT; 0.1% sodium azide) and incubated for 10 min at room temperature (RT) before centrifugation for 3 min at 1800 × g.

Next, cells were resuspended in 1 ml of spheroblasting buffer (50 mM KH_2_PO_4_/K_2_HPO_4_, pH 7.4; 0.6 M Sorbitol; 0.1 mM DTT; 0.5 mg/ml Zymolyase; 2% Glusulase) and incubated at 37°C for 30 min. After centrifugation at 1800 × g for 3 min, the spheroblasts were collected and washed twice with 0.5 ml of wash buffer (20 mM KH_2_PO_4_/K_2_HPO_4_, pH 6.5; 0.6 M Sorbitol; 1 mM MgCl_2_; 1 mM DTT; 20 mM β-glycerophosphate; 1 mM PMSF; and protease inhibitors cocktail).

Spheroblasts were then resuspended in 200 μl of the wash buffer, with 1/10 of the total cell extract retained as input. Spheroblasts were superimposed on top of 1.4 ml 18% Ficoll buffer (18% Ficoll; 20 mM KH_2_PO_4_/K_2_HPO_4_, pH 6.5; 1 mM MgCl_2_; 1 mM DTT; 20 mM β-glycerophosphate; 1 mM PMSF; 0.01% IGEPAL; and protease inhibitor cocktail), then incubated for at least 10 min on ice and centrifuged for 5 min at 5000 × g. To get the nuclei pellet, the supernatant was centrifuged a second time at 5000 × g for 5 min, followed by a final centrifugation at 16 100 × g for 20 min.

Nuclei pellets were resuspended in 200 μl of EBX buffer (50 mM HEPES/KOH, pH 7.5; 100 mM KCl; 2.5 mM MgCl_2_; 0.1 mM ZnSO_4_; 2 mM sodium fluoride; 0.5 mM Spermidine; 0.25% Triton X-100; 1 mM DTT; 20 mM β-glycerophosphate; 1 mM PMSF; and protease inhibitor cocktail) and lysed on ice for at least 10 min.

Lysates were then transferred onto 500 μl of EBX-S Buffer (EBX buffer with 30% sucrose) and centrifuged at 16 000 × g for 10 min. The resulting chromatin pellets were gently resuspended in 1 ml of EBX buffer before being centrifuged at 10 000 × g for 2 min. Finally, the chromatin samples were resuspended in 30 μl of 1.5× Bolt LDS Sample Buffer, boiled at 75°C for 10 min, and immunoblotted using the specified antibodies. Immunoblotting against PGK1 (to measure cytoplasmic contamination; Abcam, cat# ab113687 clone 22C5D8) and histone H3 (to test chromatin extraction; Invitrogen, cat# Pa5-16183) was used to confirm the quality of the fractionation.

### Cell cycle analysis by flow cytometry analysis (FACS)

Yeast cells were collected at various time points for verifying G1-arrest and cell cycle progression. At each time point, one ml of growing yeast cells was harvested by quick spin at 17 949 × g for 1 min, fixed in 1 ml of 70% ethanol, and stored at 4°C for a maximum of 2 weeks. Fixed cells were then pelleted by centrifugation at 3800 × g at 4°C for 2 min, washed once with 300 μl 50 mM sodium citrate (NaCi) pH 7.2 and centrifuged a second time at 3800 × g for 10 min. After the wash, pellets were resuspended in 250 μl of NaCi and 5 μl of RNase A (stock 10 mg/ml) was added for RNA digestion at 37°C for a minimum of 1 h. Propidium iodide staining with a final concentration of 25 μg/ml was carried out at 37°C for another minimum of 1 h. Stained samples were sonicated with five 5 s pulses, just before cell cycle analysis on the Gallios 8 Flow Cytometer (Beckman Coulter). Analyses were performed with Kaluza software.

### Flp-nick induction, chromatin immunoprecipitation (ChIP) and quantitative real-time PCR (qPCR) of *flp-H305L* and ubiquitin at the *FRT*

#### Flp-nick induction and harvesting of cells

Induction of *flp-H305L-3HA* expression was performed as described in (47) with slight modifications. Overnight yeast cultures grown in YEP–2% raffinose were diluted to OD_600_ = 0.2 and grown until log phase. Cells were then arrested in G1 by addition of 200 ng/ml α -factor for 1.5 h. Galactose was then added to final concentration of 3% for 2 h. Cells were washed twice with 20 ml cold YEP (no sugar) or YEP + α -factor if kept in G1 after. They were then either released into warm YEP- 2% glucose (YEPD) or YEPD + α -factor if not released into the cell cycle.

For *flp-H305L-3HA* ChIP, cells were not crosslinked before collection. For Ubiquitin ChIP, cells were crosslinked with 1% formaldehyde for 15 min at room temperature, quenched with 250 mM glycine and incubated for 5 min, followed by ice-cooling for at least 10 min. Before freezing, pellets were washed twice with cold 1× phosphate buffered saline (PBS) and transferred into 2 ml screw-cap tubes.

#### ChIP and qPCR

All subsequent steps were performed at 4°C or on ice, if not mentioned otherwise. Frozen pellets were resuspended in 1 ml cold FA lysis buffer (50 mM HEPEs–KOH pH 7.5; 140 mM NaCl; 1 mM EDTA; 1% Triton X-100; 0.1% sodium deoxycholate; protease inhibitor cocktail) and lysed by MagNA Lyzer (6000 rpm; 5 cycles of 30 s; 1 min on ice between runs). Extracts were recovered in a new tube and centrifuged at 13 000 × rpm for 30 min. After resuspension with 1 ml of fresh FA lysis buffer, extracts were sonicated *via* Bioruptor Twin (Diagenode) at 4°C for 20 cycles of 30 s. After centrifugation at 13 000 × rpm for 15 min, supernatant was transferred in a new tube and the amount of protein was determined by Bio-Rad protein assay (cat# 500-0006).

Each immunoprecipitation was carried out with 1 mg of protein (1/10 retained as the input) incubated overnight at 4°C under rotation, with 1 μl of anti-HA or 1 μl of anti-ubiquitin. Protein G sepharose beads were then added for 3 h, at 4°C under rotation. After incubation, beads were washed 1× with 500 μl cold FA lysis buffer, 2× with 500 μl cold FA-500 buffer (FA lysis with 500 mM NaCl), 2× with 500 μl Buffer 3 (10 mM Tris–HCl pH 8; 1 mM EDTA; 250 mM LiCl, 1% IGEPAL; 1% sodium deoxycholate) and 1x with 500 μl 1× TE (50 mM Tris–HCl pH 7.5; 10 mM EDTA). Elution was then performed twice with 100 μl elution buffer (50 mM Tris–HCl pH 7.5; 1% SDS; 10 mM EDTA) incubated at 65°C for 8 min. Eluted sample and input were treated with 0.75 mg/ml Proteinase K and incubated at 42°C for 2 h, followed by de-crosslinking overnight at 65°C.

Both IP and input were purified with the MinElute PCR Purification kit (Qiagen, cat# 28006), following manufacturer's recommendations and eluted from the column twice with 30 μl of elution buffer from the kit. DNA fragments were amplified using a CFX96 Real-Time PCR machine (Bio-Rad) and SYBR Green PCR Master Mix (Applied Biosystems, cat# 4472942) with oligonucleotide pairs listed in the List of antibodies and reagents. Each amplification was performed as a technical duplicate. White plates were used and sealed with adhesives. The CFX Manager software (Bio-Rad) was used to extract the Cycle Threshold (Ct) values. IP values were normalized to input values and to the unrelated intergenic region, where indicated.

### Purification of ubiquitinated species by inducible expression of HIS-ubiquitin

Plasmid containing HIS-Ubiquitin under a copper-inducible promoter was first transformed in yeast strains of interest.

#### Culture preparation

Cells were pre-grown in selective medium without copper overnight. The next day, cells were diluted and grown in medium supplemented with 100 μM of CuSO_4_, for HIS-Ubiquitin expression. 100 OD of cells was collected by addition of TCA to a final concentration of 5%, followed by incubation on ice for 20 min and spin at 1620 × g for 5 min. Pellet was then washed once with water and twice with acetone, and dried under vacuum.

#### Cell lysates preparation

Pellets were resuspended in 1 ml of fresh Guanidinium buffer (10 mM NaPi pH 8.0; 10 mM Tris–HCl pH 8.0; 10 mM β-mercaptoethanol; 0.1% Triton X-100; protease inhibitor cocktail (Roche), 10 mM imidazole; 0.1 mM MG132; 2.5 mg/ml NEM, mixed with 200 μl of glass beads and subjected to bead-beating in a MagNa Lyzer instrument (Roche) 6 times for 30 s at 4°C, 1 min of pause in between. Lysates were then collected in a new tube by centrifugation to get rid of the beads and further spun in a table-top centrifuge at 18 000 × g for 20 min. Supernatant was transferred in a new tube and protein concentration was determined by Bradford assay (Bio-Rad, cat# 5000006). Pulldown was performed on 6 mg of total proteins resuspend in 1 ml of Guanidinium buffer. 50 μl of input was kept and additionally mixed with 350 μl of water and 100 μl of 50% TCA, incubated at room temperature for 15 min. Inputs were then spun at 18 000 × g for 30 min at room temperature. Input pellets were washed twice with acetone and vacuum dried. 40 μl of 2× sample buffer was added and inputs were then boiled for 10 min at 98°C.

#### Ni-NTA column preparation

80 μl of Ni-NTA beads suspension (Qiagen, cat# 1018244) was used per pulldown. Beads were washed twice with water and equilibrated once with Guanidinium buffer. After the last wash, beads were resuspended in 120 μl of Guanidinium buffer per sample before being added to the lysates. Pulldown was performed for 2 h at room temperature, under rotation.

#### Pulldown and elution

Tubes were spun at 450 × g for 2 min at room temperature. Supernatant containing the unbound proteins was removed and beads containing ubiquitinated proteins were washed once with 1 ml Guanidinium buffer and 3 times with 1 ml urea buffer (10 mM NaPi pH 6.4; 10 mM Tris–HCl pH6.4; 8 M urea; 10 mM β-mercaptoethanol; 0.1% Triton X-100). Elution of ubiquitinated species from the beads was performed by addition of 40 μl of 2× sample buffer and boiled at 98°C for 5 min. The following antibodies were used for western blot analyses: anti-polyhistidine (Sigma, cat# H1029 – lot 034M4777V); anti-Pgk1 (Abcam, cat# ab113687 clone 22C5D8); anti-MYC (homemade).

### ChEC-seq of Rtt101

#### Sample preparation for ChEC

Experiments were performed as described in (48) with minor modifications. Yeast strains were cultured in YEP medium supplemented with 2% raffinose overnight. Cells were diluted to OD_600_ = 0.25 in 130 ml of YEP-2% raffinose for 3 h. Then, 50 ml was separated for the induction of *flp-H305L* expression with 3% galactose for 2 h.

For each condition, 50 ml of cultures were harvested at room temperature at 1500 × g for 30 s. Cells were quickly resuspended in 1 ml of Buffer A (15 mM Tris pH 7.5; 80 mM KCl; 0.1 mM EGTA; 0.2 mM spermine; 0.5 mM spermidine; 1 mM PMSF; protease inhibitor cocktail), transferred into 2 ml tubes and pelleted at room temperature at 1500 × g for 30 s. Cells were permeabilized by resuspending in 693 μl of Buffer A supplemented with 7 μl of 10% digitonin and incubated at 30°C with shaking for 5 min. An aliquot of 100 μl was separated and kept as a negative control (no MNase digestion).

Then, 3.5 μl of 1 M CaCl_2_ (5 mM final) was added and tubes were immediately placed at 30°C to start MNase cleavage. An aliquot of 100 μl was taken at 30 s and immediately mixed with 100 μl of Stop Buffer (400 mM NaCl; 20 mM EDTA; 4 mM EGTA; 0.1% SDS). The procedure was repeated for later time points.

Once all time points were collected, cells were treated with 4 μl of 20 mg/ml proteinase K and incubated 30 min at 55°C. DNA was then extracted by the addition of 200 μl of phenol:chloroform:isoamyl alcohol followed by 5 min centrifugation at maximum speed at room temperature. Then, 150 μl of the aqueous phase was transferred into a new tube, and DNA was precipitated by the addition of 1.5 μl of 20 mg/ml glycogen and 500 μl of 100 % ethanol and incubated at –20°C overnight. Precipitated DNA was centrifuged at maximum speed at 4°C for 10 min. DNA pellets were washed with 1 ml of 70% ethanol and air-dried for 5 min. Dried pellets were resuspended in 48 μl of 10 mM Tris–HCl pH 8.0 and 2 μl of 10 mg/ml RNase A was added for RNA digestion at 37°C for 30 min.

For size selection, 100 μl of AmPure XP beads (Beckman Coulter, cat# A63881) was added and the bead:DNA mixture was incubated at room temperature for 5 min. Beads were collected by placing tubes on a magnetic rack, and the supernatant was transferred to a new tube containing 55 μl of 10 mM Tris–HCl pH 8.0 and 4 μl of 5 M NaCl. Another DNA extraction and precipitation was performed as described above.

DNA pellets were washed and resuspended in 50 μl of ultrapure water. The concentration was determined using a QuBit dsDNA high-sensitivity assay (Invitrogen, cat# Q32854).

Sequencing libraries were prepared using NEBnext Ultra DNA library prep kit for Illumina (NEB, cat# E7370L) following manufacturer's protocol that does not rely on size selection. Samples were sequenced using a paired-end approach at the iGE3 genomics sequencing platform of the University of Geneva.

#### ChEC-seq mapping and analyses

Adapters were removed from the paired-end reads using the Trim Galore! function with default options from the Galaxy server (49,50). Trimmed paired-end reads were then aligned to the modified version of the sacCer3 (called sacCer3-Flp-nick and shared as a fasta file) containing an insertion of *FRT* sites together with a *URA3* marker in between the *ROG3* and *ATG18* coding genes. The bamCoverage function of DeepTools 2.0 (51) was used to create bigWig density files with the following options: a bin size of 1bp, counts per million (cpm) normalization with the exclusion of the chromosome M, 20 as a minimum Phred quality score and centering regions with respect to the fragment length limited to 200 bp. BigWig files were visualized using Integrate Genome Browser (IGB) (52,53). The average of the two replicates and the differences between the ChEC signal in Galactose and Raffinose-containing media were produced using the bigWigCompare function of DeepTools 2.0.

### ChIP-sequencing analysis of Rtt101 and DNA Pol 2

#### Sample preparation for ChIP

Yeast cells were grown in YEPD until exponential phase, and synchronized in G1 phase by addition of α-factor (PRIMM; cat# 201307–00007) for 2 h. After two washes, cells were released into the cell cycle in fresh YEPD medium without drugs, or supplemented with 200 mM of HU. Cell cycle progression was monitored by FACS analysis. Yeast cells were fixed with formaldehyde and collected for ChIP as described in Flp-nick induction, chromatin immunoprecipitation (ChIP) and quantitative real-time PCR (qPCR).

The DNA concentration was measured using Qubit dsDNA HS kit (Invitrogen, cat# Q32854). Sequencing libraries were prepared using NEBnext Ultra DNA library prep kit for Illumina (NEB, cat# E7370L) following manufacturer's guidelines. Finally, samples were sequenced at the iGE3 genomic platform of the University of Geneva.

#### ChIP-seq mapping and analyses

After adapters removal using Trim Galore!, reads were aligned to the sacCer3 genome via Bowtie2 (54) through the Galaxy server. BigWig density files were then produced using the bamCoverage function with the following options: a bin size of 250 smoothed by a rolling window of 1000 bp, cpm normalization with the exclusion of the chrM, centering fragments after their artificial extension to 250 bp and a minimal Phred quality score of 20. Differential densities between time points in S-phase and G1-phase were processed through the bigwigCompare function. Metagene plots were made using the computeMatrix and plotProfile commands. The list of ARS Consensus Sequences (ACSs, listed in Supplementary Table S2) was retrieved from (55) and their orientation was taken into account. The 40 early ARSs were defined through a *k*-means clustering based on the DNA Pol 2 signal 10 kb around the ACSs. The median *Z*-score was finally calculated on the 30 kb-window around ACSs.

### Quantification and statistical analysis

GraphPad Prism 8 software was used to generate graphs and statistical analyses. Statistical tests and samples sizes are mentioned in the figure legends.

## Results

### The cullin Rtt101 participates in resistance to Top1-DNA adducts

DPCs can be classified as enzymatic or non-enzymatic depending on the characteristics of the crosslinked protein and the mechanism triggering their formation (6). For instance, Topoisomerase 1 (Top1, human TOP1) can become covalently trapped on the DNA upon abortive enzymatic reaction, thus forming a Top1-DNA crosslink, or Top1cc. Likewise, Top2ccs are considered as enzymatic DPCs. In yeast, Top1-DNA adducts (Top1ccs) are one of the most investigated DPC types. Their clearance involves the action of both the protease Wss1 and the phosphodiesterase Tdp1. Tdp1 is a specialized enzyme that directly hydrolyzes the covalent bond formed between Top1 and the DNA (56). Hence, a double mutant *tdp1Δ wss1Δ* accumulates high loads of Top1ccs under unchallenged conditions, and exhibits hypersensitivity to their induction by the Top1-poison camptothecin (CPT) (13).

To identify new regulatory components involved in DPC signaling, we analyzed the previously published (24) genetic network obtained *via* a SATAY transposon screen (57) of *tdp1-degron* + auxin *wss1Δ* (hereafter denoted *tdp1wss1*) which is defective in Top1cc processing (Figure 1A and B). The *tdp1-degron* (auxin-inducible degron, (58,59)) is used for rapid auxin-mediated degradation of Tdp1, enabling the investigation of the *tdp1Δ wss1Δ* mutant, which would otherwise be nearly nonviable. The screen reveals possible synthetic lethal interactors (less transposed; Figure 1B, left) or suppressors (more transposed; Figure 1B - right) of *tdp1wss1*. Among the strong genetic suppressors of *tdp1wss1* found in the screen is *TOP1* (Figure 1B), which was previously shown to be the main detrimental factor for the growth of the *tdp1Δ wss1Δ* mutant, with Top1ccs being the main source of stress for this mutant strain (13,60). This served as a positive control for the experiment, thus validating previous observations. We then aimed to identify genes associated with post-translational ubiquitin modification, which is presumed to be important for Top1ccs resistance.

Rtt101, one of the three cullins found in budding yeast (61), is the core component of multiple E3 ubiquitin ligase complexes, each specific to different sets of substrates depending on the associated receptor protein (Figure 1C) (42,43). Intriguingly, the *RTT101* gene was depleted of transposition events upon loss of Wss1 and Tdp1 enzymes (Figure 1B and Supplementary Figure S1A). Moreover, genes constituting the known components of Rtt101-based ubiquitin ligase complexes (Figure 1C) also showed diminished transposition events in the absence of Wss1 and Tdp1 (Figure 1B). This implies that this set of genes is probably relevant for Top1cc processing, in addition to Wss1 and Tdp1 (Figure 1D).

Subsequent genetic analyses confirmed that the absence of Rtt101 exacerbated the growth impairment of *tdp1Δ wss1Δ* spores (Figure 1E and Supplementary Figure S1B, square and round shapes). Mms1, which functions as the linker for Rtt101-based ligases (42,43) and facilitates interactions with substrate-specific adaptors, similarly exhibited detrimental effects upon its deletion in *tdp1Δ wss1Δ* spores (Supplementary Figure S1C). This indicates the critical role likely played by multiple elements within the ligase complex. Furthermore, the deletion of either *RTT101* or *MMS1* in *tdp1wss1* increased susceptibility to the stabilization of Top1cc by the targeted drug CPT (Figure 1F and Supplementary Figure S1D, lines 7–8). Interestingly and in line with previous studies (41), the *rtt101Δ* single deletion mutant already displayed sensitivity to CPT (Figure 1F, line 2), suggesting its potential significance in repairing Top1cc lesions. Thus, we postulated that the Rtt101^Mms1^ ligase complex is required for resistance against Top1ccs.

In mammals, the functional counterpart of Rtt101, known as CUL4B, plays a crucial role in resistance to CPT and in mediating TOP1 ubiquitination and degradation following exposure to the drug (40). Consistently, the absence of Rtt101 in yeast significantly impacted CPT-induced degradation of Top1 (Figure 1G). As CPT traps Top1 on chromatin (62), we assessed the behavior of chromatin-associated Top1 in response to the *rtt101Δ* mutation. As anticipated, Top1 was more persistent on chromatin in *rtt101Δ* cells subjected to CPT treatment (Figure 1H). These findings support the idea that Rtt101 mediates Top1ccs resistance and removal from chromatin.

### Rtt101 provides resistance to a broad range of DPCs

We speculated that Rtt101 might have a broader function beyond specifically addressing Top1ccs, and could potentially play a rather general role in protecting cells against enzymatic DPCs. To test this idea, we first combined a null mutant of Rtt101 with either *wss1Δ* or *tdp1Δ*. As the cell wall of yeast is not permeable, we took advantage of the *12geneΔ0HSR* mutant (63) to reveal cellular sensitivities towards CPT and etoposide (ETO), respectively trapping Top1 and Top2 on the DNA.

As previously observed, both *wss1Δ* and *rtt101Δ* mutants were sensitive to Top1ccs (Supplementary Figure S1E, lines 2–3; (24,41,64)), although *rtt101Δ* presented higher sensitivity. Interestingly, in this *12geneΔ0HSR* background, we were able to reveal the hypersensitivity of the *rtt101Δ* mutant towards Top2ccs (Supplementary Figure S1E, line 2). This finding aligns with previous observations made for its binding partner, Mms22 (65). A double mutant *rtt101Δ wss1Δ* displayed a strong synergistic effect on both topoisomerase-trapping drugs (Supplementary Figure S1E, line 5), emphasizing a critical role of both enzymes in limiting the toxicity of enzymatic-induced DPCs.

Given that Top1ccs and Top2ccs are associated with single- or double-strand DNA breaks (66), we wanted to examine the resistance of the *rtt101Δ* mutant to non-specific crosslinking chemicals, such as formaldehyde (FA) and hydroxyurea (HU) (Figure 1I). Analogous to observations made for enzymatic DPCs (Figure 1F and Supplementary Figure S1E), the viability of the *rtt101Δ* mutant was greatly impacted upon non-enzymatic DPC induction with FA and HU compared to a WT strain (Figure 1I, lines 1–2).

We also confirmed that the accumulation of Top1 was the main cause of CPT-induced sensitivity in *rtt101Δ* cells (Figure 1I, lines 2 and 4), but not the cause of cell death in the presence of HU and FA. Deletion of *TOP1* in *rtt101Δ tdp1, tdp1wss1* and *rtt101Δ tdp1wss1* cells also rescues CPT but not HU sensitivity (Supplementary Figure S1F, lines 3–4, 7–8), supporting the notion that Top1ccs represent only a fraction of DPCs potentially targeted by Rtt101.

Notably, while we employed HU as a potential DPC inducer, this compound is more recognized for its capacity to induce replication stress (67). Nonetheless, although HU has not been linked to the formation of DPCs, it creates free radicals (68,69), themselves responsible for the creation of DPCs (70). Therefore, our data support the fact that Rtt101 may also promote fork progression in general and help to tolerate replication stress, as already proposed (45).

### A complete and functional Rtt101^Mms1^ complex is required for DPC resistance

**Figure 2.**
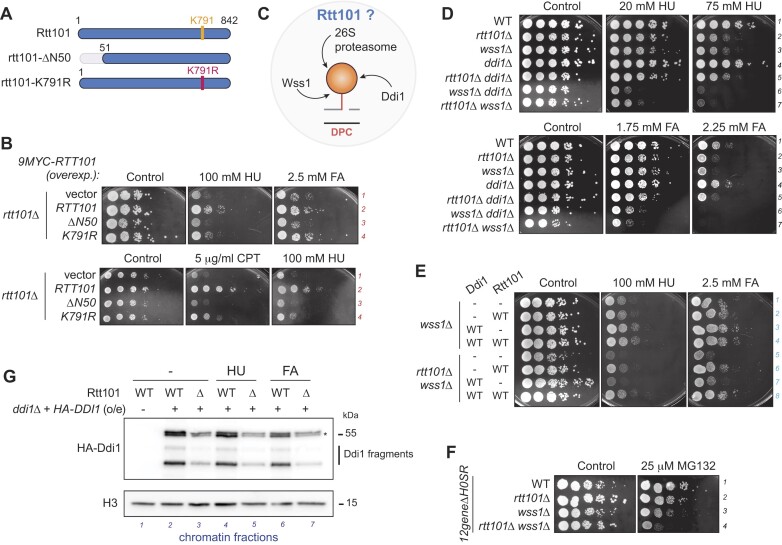
Rtt101 confers resistance to a broad range of DPCs in a pathway dependent on Ddi1. (**A**) Schematic representation of the Rtt101 cullin and its variants. The lysine 791 (K791) is modified by ubiquitination or neddylation. When mutated, K791R is unable to undergo modification. The N-terminal domain mediates interaction with the linker protein Mms1 (N50 domain). These constructs are used in (B) and are all tagged with 9MYC on their N-terminal side. (**B**) A complete and functional cullin ligase complex is important for resistance against DPCs. Activation of Rtt101 or interaction with the linker Mms1 is crucial. The *rtt101Δ* mutant was complemented with plasmids overexpressing Rtt101 wild-type (WT) or variants *(ΔN50, K791R*) (shown in (A)). Cells were grown in liquid selective medium to avoid loss of plasmids and plated on 100 mM hydroxyurea (HU) and 2.5 mM formaldehyde (FA) (top panel), or on 5 μg/ml CPT and 100 mM HU (bottom panel). Plates were incubated for 2 days at 30°C. See Supplementary Figure S2A for protein levels. (**C**) The 26S proteasome, Wss1 and Ddi1 are the known proteases actively repairing DPCs. How does Rtt101 engage in genetic interactions with these pathways? (**D**) Genetic interactions of Rtt101, Wss1 and Ddi1 in resistance against DPCs. Rtt101 and Ddi1 are genetically linked, while Rtt101 and Wss1 work in parallel pathways for resistance towards DPCs induced chemically by HU and FA. Cells were grown in YEPD and spotted on a medium supplemented with indicated concentrations of HU and FA. Plates were incubated for 2 days at 30°C. (**E**) Rtt101 and Ddi1 are working in the same pathway. Ddi1 is not proficient in alleviating *wss1Δ* phenotype on HU and FA in the absence of the cullin Rtt101. Mutants *wss1Δ* or *rtt101Δ wss1Δ* were co-transformed with plasmids overexpressing wild-type (WT) HA-Ddi1, 9MYC-Rtt101, or both. – indicates transformation with the corresponding empty vector. Experiment was performed as in (B). See Supplementary Figure S2B for protein levels. (**F**) Rtt101 works in a pathway parallel to the 26S proteasome. The *12geneΔH0SR* genetic background is used to allow efficient uptake of MG132 (26S proteasome inhibitor). Experiment was performed as in (D). Plates were incubated for 3 days at 30°C. (**G**) The presence of Ddi1 on chromatin partially depends on the ubiquitin ligase Rtt101. Cells were subjected for 2 h to 200 mM HU treatment (lanes 4–5) or were released for 2 h from a 15 min treatment of 40 mM FA (lanes 6 -7). Chromatin fractions were isolated from indicated mutants complemented with overexpressed HA-Ddi1 construct. Levels on chromatin were compared by immunoblotting, and histone H3 was used as a loading control. Star indicates full-length Ddi1.

Rtt101 forms a complex with its linker protein, Mms1, through its N-terminal domain (42). Additionally, Rtt101 undergoes post-translational modifications such as neddylation or ubiquitination at lysine K791 (64,71). To elucidate the role of Mms1 binding and the significance of K791 modification in the function of Rtt101 in DPC repair, we conducted complementation assays of *RTT101* inactivation using plasmids expressing mutated variants of Rtt101 (Figure 2A, B and Supplementary Figure S2A). Our results revealed that the interaction between Rtt101 and Mms1 is indispensable for growth on CPT, HU and FA (Figure 2B, lines 3). This observation underscores the necessity of a complete cullin complex for resistance to DPCs. Interestingly, we also observed that an Rtt101 mutant in which K791 was substituted with arginine (K791R) only partially restored resistance to the same compounds (Figure 2B, lines 4), suggesting that K791 modification is significant for Rtt101 functionality towards DPCs.

Overall, these data argue that the Rtt101^Mms1^ ubiquitin ligase complex may target a broad spectrum of DPCs for repair, regardless of the adduct type.

### Rtt101 and the ubiquitin-dependent DPC protease Ddi1 act in the same pathway

Proteases play pivotal roles in the repair of DPCs. In budding yeast, the key DPC proteases identified include Wss1, Ddi1 and the 26S proteasome (Figure 2C). Given the role of ubiquitin in orchestrating DPC repair processes, we sought to explore the genetic interactions between *RTT101* and the corresponding protease genes. In *rtt101Δ* cells, we additionally deleted *WSS1* or *DDI1* (Figure 2D). Consistent with previous findings, the aspartic protease Ddi1 operates in parallel to the DNA-dependent protease Wss1 (Figure 2D; lines 3–4 and 6) (23,24,72). Similarly, Rtt101 is engaged in genetic pathways functioning in parallel to Wss1 (Supplementary Figure S1E), also under HU and FA stress (Figure 2D, lines 7). In contrast, the simultaneous deletion of both *RTT101* and *DDI1* did not decrease cellular resistance to FA or HU, suggesting that Rtt101 and Ddi1 function within the same pathway (Figure 2D, lines 5).

Strikingly, while Ddi1 overexpression can compensate for the loss of Wss1 and Tdp1 (24), this compensatory mechanism relies on the presence of Rtt101 (Figure 2E and Supplementary Figure S2B). Indeed, overexpression of Ddi1 in Rtt101-depleted cells did not rescue the phenotype of *wss1Δ* on HU and FA stress (Figure 2E, lines 3, 7–8), despite unaffected protein expression levels (Supplementary Figure S2B). Similarly, in the *tdp1wss1* background, Ddi1 overexpression mediated HU resistance via Rtt101, and only partially restored resistance to CPT in the absence of Rtt101 (Supplementary Figure S2C, lines 2 and 4; Supplementary Figure S2D). Additionally, genetic analyses combining the protease dead *ddi1-D220A* and *rtt101-K791R* variants confirmed that a WT-Rtt101 is required for the compensation mediated by WT-Ddi1. In contrast to the overexpression of Ddi1 capable of compensating the loss of Wss1 [(Figure 2E, line 3), (24)], overexpressed Ddi1 does not compensate the loss of Rtt101 (Supplementary Figure S2E - line 2 and S2F), emphasizing the intrinsic connection between Rtt101 and Ddi1. It is therefore possible that Rtt101 works upstream of Ddi1.

Last, codeletion of *rtt101Δ* and *wss1Δ* revealed an additive phenotype when exposed to proteasome inhibition with MG132 (Figure 2F, line 4), suggesting that the proteasome operates within another Wss1- and Rtt101-independent pathway.

Altogether, the above results indicate that Rtt101, together with Ddi1, counteracts a diverse range of DPCs in a manner independent of Wss1 and the 26S proteasome.

### The chromatin localization of Ddi1 depends on Rtt101

The observations above support the notion of a functional relationship between Rtt101 and Ddi1. Given that Ddi1 is enriched on chromatin (24) and targeted to its substrates in a ubiquitin-dependent manner (73), we then considered a role for Rtt101 in recruiting Ddi1 to chromatin. To test this, we monitored chromatin-associated Ddi1 under untreated and stress conditions (Figure 2G). Interestingly, loss of Rtt101 affects the proper localization of Ddi1 on chromatin in all tested conditions (Figure 2G, lanes 3, 5 and 7), despite comparable Ddi1 levels between WT and *rtt101Δ* cells (Supplementary Figure S2F). This result reveals that a fraction of Ddi1 depends on Rtt101 for its recruitment to chromatin, indicating that Rtt101 likely acts upstream of Ddi1.

### Loss of *RTT101* delays removal of crosslinked Flp

**Figure 3.**
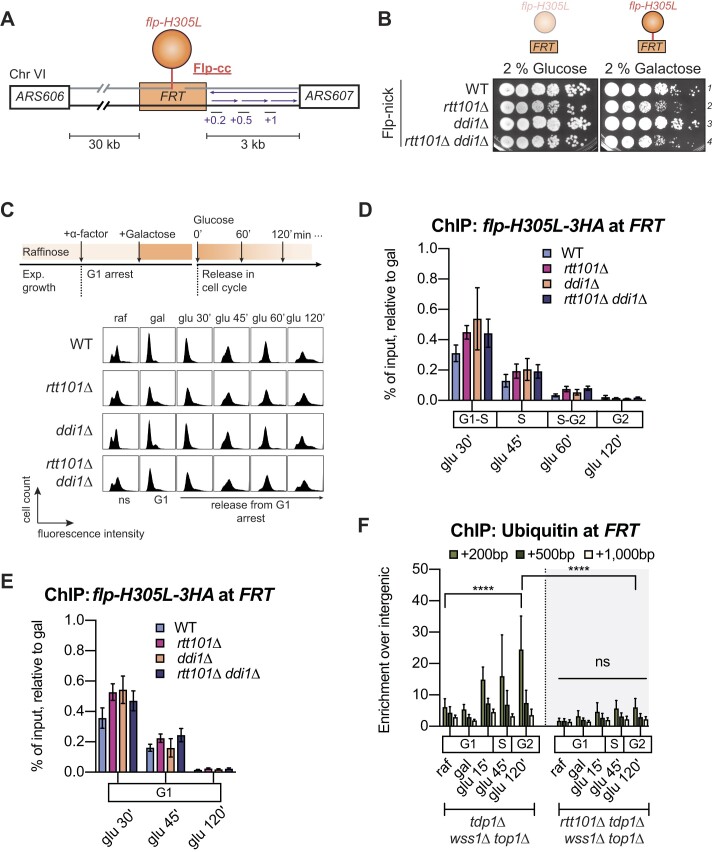
Absence of Rtt101 delays removal of DPC from DNA. (**A**) Scheme depicting features of the Flp-nick system initially described in (47). The mutant Flp recombinase *flp-H305L* recognizes and binds the *FRT* locus to create a Top1cc-like DPC (called Flp-cc). The *FRT* site was introduced into the yeast genome on chromosome VI between *ARS606* and *ARS607*. The *flp-H305L* is expressed from a galactose-inducible promoter and tagged with 3HA. Primers used for qPCR analysis are shown in purple, at 0.2, 0.5 and 1 kb away from the *FRT* site. (**B**) Yeast cells are sensitive to Flp-cc in the absence of Rtt101, and Rtt101-Ddi1 are epistatic. Yeast cells were grown in YEP liquid medium containing 2% raffinose as the source of sugar and then spotted on plates containing glucose or galactose. *flp-H305L* is expressed and creates a Flp-cc only with galactose as the source of sugar. Plates were incubated for 2 days at 30°C. (**C**) Experimental design applied in (**D**) for measuring Flp-cc kinetics at the *FRT* locus. Cells were first grown in 2% raffinose (raf), and then synchronized in G1 with α-factor for 2 h. *flp-H305L* was induced by the addition of 3 % galactose (gal) for 2 h in the presence of α-factor. Cells were then transferred in glucose to repress *flp-H305L* expression and released into the cell cycle. Cell cycle analysis was monitored by fluorescence-activated cell sorting (FACS). (D) Loss of *RTT101* delays Flp-cc kinetics during some cell cycle phases. The percentage of input for each genotype was normalized to maximum galactose induction. Yeast strains were grown as shown in (C). Levels of *flp-H305L-3HA* at the *FRT* locus were assessed by ChIP and qPCR without formaldehyde crosslinking. qPCR primers align 0.2 kb downstream of *FRT*, as shown in (A). Data are presented as mean (SEM) of the percentage of input of at least three independent biological replicates. In addition to indicated mutations, all strains are in *bar1Δ flp-H305L-3HA* genetic background. (**E**) Rtt101 acts on the removal of Flp-cc in G1. Yeast strains were grown as in (C) but kept in α-factor when transferred to glucose. Data were analyzed as described in (D). Graph represents the mean (SEM) of at least six independent biological replicates. Strains are the same as in (D). (**F**) Ubiquitination of the *FRT* locus depends on Rtt101. Cells were grown as described in (C). Ubiquitin antibody was used for ChIP-qPCR analysis following formaldehyde crosslinking. The graph shows qPCR signals (over the unrelated intergenic region) as mean (SD) of three independent biological replicates. **** *P* < 0.0001; ns, non-significant (two-way ANOVA, Tukey's multiple comparison test). In addition to indicated mutations, strains are *bar1Δ* background.

To specifically decipher the underlying molecular events linked to DPC formation and clearance, we employed the *in vivo* Flippase-nick system (Flp-nick; Figure 3A, (47)) which mimics a Top1cc-like crosslink at a single *FRT* (flippase recognition target) locus artificially introduced on chromosome VI of the yeast genome. This system relies on the galactose-dependent overproduction of a mutant Flp recombinase *flp-H305L*, which becomes covalently stabilized in a Top1cc-like structure when attempting to cleave its *FRT* target site, thereby creating what we refer to as ‘Flp-cc’.

When plated on galactose-enriched medium, *rtt101Δ* cells exhibited sensitivity to Flp-cc compared to WT-like cells (Figure 3B, lines 1–2). As observed with genetic analyses on drugs, Rtt101 and Ddi1 are epistatic also regarding the Flp-cc (Figure 3B, lines 2–4) when spotted on solid medium, emphasizing the toxicity of a single Flp-cc in the yeast genome.

The Flp-nick system provides an effective molecular tool to follow DPC repair by looking at Flp-cc repair kinetics. To achieve this, we induced the expression of the mutant *flp-H305L* in G1-arrested cells with galactose, which will form a crosslinked Flp-cc at the *FRT* locus. Flp transcription was then repressed by switching the source of sugar to glucose, and cells were released into the cell cycle (Figure 3C). We monitored Flp-cc removal from the DNA in the presence or absence of Rtt101 (Figure 3D) by ChIP-qPCR. As observed before (24,32,47,74), Flp-cc was rapidly eliminated from the *FRT* in WT-like cells (Figure 3D), with only around 30% of the initial Flp-cc amount remaining on the DNA after 30 min (Figure 3D, glu 30′). In contrast, the *rtt101Δ, ddi1Δ and rtt101Δ ddi1Δ* mutants displayed a higher retention of Flp-cc compared to WT when released into the cell cycle (Figure 3D, glu 30′ and glu 45′). After replication, *rtt101Δ* and *rtt101Δ ddi1Δ* cells still conserved more Flp-cc on the DNA compared to WT cells, while *ddi1Δ* cells resemble a WT situation (Figure 3D, glu 60′). Surprisingly, despite the dependence of replicating cells on Rtt101, the Flp-cc was eventually evicted from the DNA and no discernable difference between the mutant and WT cells was observed after 120 min of glucose release (Figure 3D, glu 120′). Although the deletion of *RTT101* delays repair in the early stages of the cell cycle such as replication, it eventually occurs through an unknown mechanism. One straightforward interpretation of this result is that, while not essential, Rtt101 is important in replicating cells encountering a Flp-cc. Potentially, other repair pathways could take care of the remaining Flp-cc in G2-phase.

To test the hypothesis that Rtt101 may be required primarily during the S-phase of the cell cycle, we used the Flp-nick system in non-replicating cells arrested in G1 in which we hypothesized Rtt101 is dispensable. Similar to previous experiments, crosslinking of *flp-H305L* was induced in G1 by galactose, but cells were then transferred to glucose supplemented with α-factor to prevent release into the cell cycle during Flp-cc repair (Figure 3E and Supplementary Figure S3A). Unexpectedly, the *rtt101Δ* mutant showed slower removal of Flp-cc from the DNA in G1-arrested cells (Figure 3E), suggesting that Rtt101 is important throughout the cell cycle and not exclusively during replication. Observations for *ddi1Δ* and *rtt101Δ ddi1Δ* mutants were also similar to those of S-phase (Figure 3D and E).

Overall, Flp-cc kinetics of removal resemble genetic observations, with *rtt101Δ* leading to sensitivity and higher retention of Flp-cc on the DNA, and *rtt101Δ* and *ddi1Δ* showing no to little additivity towards Flp-cc retention. The *ddi1Δ* mutant is closer to the WT phenotype, despite having a slight delay in Flp-cc removal both in G1 and S-phase.

### Rtt101 ubiquitinates the vicinity of crosslinked Flp

As Rtt101 is a ubiquitin ligase, we also assessed ubiquitin levels at the *FRT* locus in the presence or absence of Rtt101. Since increased ubiquitin levels at the *FRT* cannot be revealed under WT-like conditions, we examined the *tdp1Δ wss1Δ top1Δ* mutant which we previously used to demonstrate significant ubiquitin enrichment upon Flp-cc formation (32). Following Flp-cc induction, the S-phase release of cells lacking Wss1 and Tdp1 correlated with a great ubiquitin enrichment in the vicinity of the Flp-cc (Figure 3F). Notably, loss of Rtt101 led to a significant decrease in the ubiquitin signal. Consistent with the role of Rtt101 in G1 (Figure 3E), the decrease in ubiquitin levels was already evident in G1-arrested cells (Figure 3F, gal time point).

In mammals, CUL4B ubiquitinates TOP1 (40,75). We reasoned that Rtt101 could ubiquitinate the adduct *per se*. As the Flp-nick system mimics a Top1cc, we tested this assumption by looking at ubiquitinated-Top1 levels following CPT treatment. The ubiquitination assay relies on the copper-inducible expression of exogenous His_6_-Ubiquitin carried on by a plasmid (Supplementary Figure S3B). This allows the purification of ubiquitinated species by using the Ni-NTA resin. However, this approach did not allow to detect an effect of Rtt101 on Top1 ubiquitination (Supplementary Figure S3C). Surprisingly, the CPT treatment did not influence the levels of Top1 ubiquitination in WT cells as well (Supplementary Figure S3C, lines 1 and 5). It is possible that the conditions and the assay used to reveal ubiquitination of chromatin-bound proteins in response to CPT are not optimal. We cannot exclude that Rtt101 may ubiquitinate Top1ccs or a factor present at DPCs involved in sensing and repair.

Collectively, these data indicate that Rtt101 contributes to the elimination of Flp-cc from DNA by ubiquitination at or around DPC sites.

### Rtt101 is specifically recruited to Flp crosslinks

**Figure 4.**
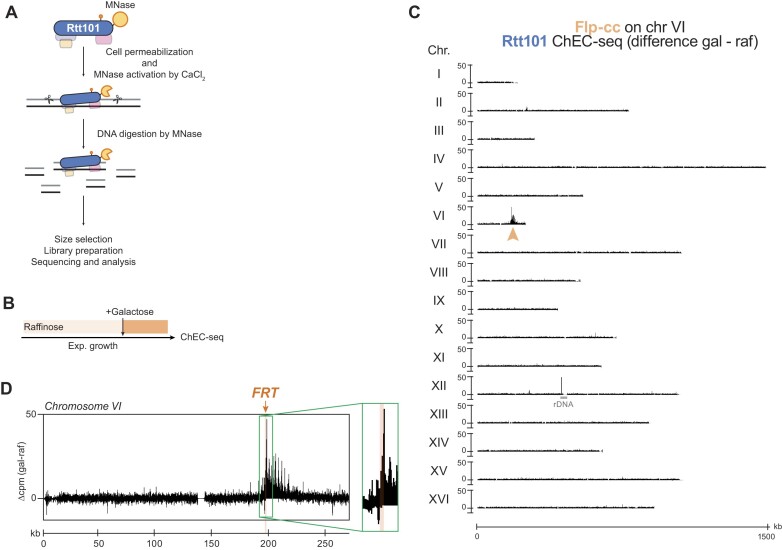
Rtt101 is recruited to Flp-cc. (**A**) Outline of the *in vivo* chromatin endogenous cleavage (ChEC) coupled with high-throughput sequencing (ChEC-seq) (77,78). Rtt101 is C-terminally tagged with MNase at its endogenous locus. Living yeast cells were collected and permeabilized with digitonin. Activation of MNase was induced with CaCl_2_, resulting in DNA cleavage and generation of fragments bound to Rtt101. Size selection was performed prior to preparation of sequencing libraries to enrich for smaller DNA fragments. (**B**) Experimental design applied in (**C**) and (**D**) for the ChEC-seq of Rtt101. Asynchronous cells were grown in 2 % raffinose and *flp-H305L* was then induced by the addition of 3 % galactose for 2 h. Living cells were pelleted and subjected to ChEC preparation as outlined in (A). Strains are in Flp background. (C) Rtt101 is enriched at Flp-cc. Genome-wide mapping of Rtt101 binding by ChEC-seq with Rtt101-MNase induced for 30 seconds. Flp-cc on chromosome VI was induced by galactose (location is indicated by the orange arrow). Data are presented as the mean of 2 replicates of (Galactose – Raffinose) difference in cpm. The rDNA locus on chromosome XII contains 100–200 repeats of the rDNA. (D) Snapshot of Rtt101 ChEC-seq signals along chromosome VI. Location of the *FRT* site is indicated by the orange arrow in the green box (see Supplementary Figure S4A for the snapshot of each individual replicate).

If the action of Rtt101 is direct, and considering its known recruitment to chromatin (76), we postulated that Rtt101 will be enriched at DPC sites. In light of its aforementioned role in Flp-cc removal (Figure 3D and E), we sought to investigate whether Rtt101 chromatin binding is increased at the *FRT* site when Flp is crosslinked by using resolutive and precise high-throughput Chromatin Endogenous Cleavage (ChEC-seq) (77,78). ChEC-seq (Figure 4A) relies on the fusion of the MNase enzyme to the protein of interest, i.e. Rtt101. MNase-dependent DNA cleavage is activated by calcium for a brief period of time, followed by selection of DNA fragments protected by Rtt101 and sequencing to reveal the footprint of Rtt101 within the genome. We performed ChEC-seq of Rtt101 in asynchronous WT-like Flp-nick strains cultured in the presence and absence of galactose, thus inducing or not Flp-cc (Figure 4B).

Through ChEC-seq analysis, we generated a differential Gal – Raf (Flp-cc – no Flp-cc) binding map of Rtt101 across the yeast genome containing the *FRT* site (Figure 4C). Notably, we observed a distinct enrichment of Rtt101 on chromosome VI (orange arrow), which contains the *FRT* site. Indeed, visualizing the ChEC-seq signal of Rtt101 on chromosome VI (Figure 4D and Supplementary Figure S4A) revealed specific colocalization of Rtt101 with the *FRT* site and, consequently, Flp-cc. This analysis does not imply that Rtt101 is exclusively recruited to chromatin in the presence of a DPC. Rather, it indicates that Rtt101 becomes concentrated at DPC sites. Of note, we also observed an enrichment of Rtt101 at the rDNA locus suggesting a putative role in rDNA processes (Figure 4C, chromosome XII).

Hence, these observations allow to conclude that Rtt101 is recruited to the crosslinked Flp, and strongly suggest that it may be similarly recruited to DPCs in general.

### Rtt101 travels with the DNA replication machinery

The linker protein Mms1 and the receptor Mms22 stabilize replisomes during replication stress (79). In addition, the binding partner Mms22 was also shown to associate with replisomes in S-phase (45), even in unchallenged conditions. Moreover, Rtt101 promotes fork progression through DNA damage (41). We therefore postulated that a fraction of the Rtt101 pool may travel with the replisome and provide a sensing mechanism for DPCs.

**Figure 5.**
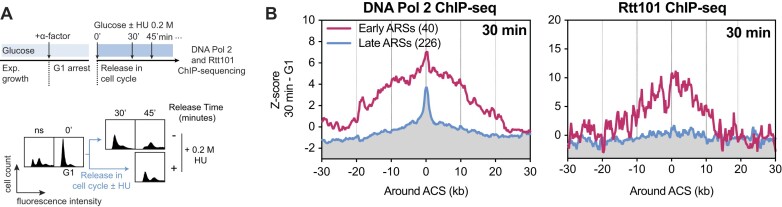
Rtt101 canonically travels with the replisome. (**A**) Experimental scheme applied in (B). Exponentially growing cells were arrested in G1-phase of the cell cycle with α-factor. Cells were then released from G1-arrest in liquid YEPD supplemented or not with 200 mM of hydroxyurea (HU). Samples were collected at different time points for FACS (bottom panel) and ChIP-sequencing analysis of DNA Polymerase 2 (DNA Pol 2; replisome component; Pol2-5FLAG) or Rtt101-3HA. ns, non-synchronized; HU, hydroxyurea. (**B**) Rtt101 and DNA Pol 2 present the same pattern on DNA. Metagene plots depicting the differential densities of DNA Pol 2 and Rtt101 binding to chromatin in S-phase (30 min) minus their respective binding in G1-phase (30 min – G1). Plots were centered on the oriented ACS (ARS consensus sequence), and separate early ARSs (*n* = 40) and late ARSs (*n* = 226). See Supplementary Figure S4B for another replicate. See Supplementary Figure S4C for analysis under HU stress.

To address this possibility, we used high-throughput sequencing to analyze the chromatin distribution of the replisome-associated component DNA Polymerase 2 (DNA Pol 2) and Rtt101 in WT cells under unchallenged conditions or replication stress. Briefly, cells were arrested in G1-phase and released into the cell cycle in the presence or absence of HU (Figure 5A). To clearly visualize the progression of the replication forks, enrichments at the indicated time points were normalized to enrichment in G1 (Figure 5B). First, analysis of sequencing reads associated with DNA Pol 2 showed that early replicating sequences (ARS) display a higher enrichment of DNA Pol 2 than late ARS at 30 min (Figure 5B and Supplementary Figure S4B, left panel), corresponding to entry into S-phase (Figure 5B). Second, Rtt101 follows the same pattern and is also significantly more present on early ARS than late ARS at 30 min (Figure 5B and Supplementary Figure S4B, right panel). Additionally, both DNA Pol 2 and Rtt101 are more enriched at the replication origin centers (Figure 5B), suggesting a similar distribution of DNA Pol 2 and Rtt101 at the beginning of S-phase.

Interestingly, cells subjected to HU replication stress also presented similar distributions of Rtt101 and DNA Pol 2, with a significant difference in enrichment between early and late replication origins (Supplementary Figure S4C). Additionally, analysis of the DNA Pol 2 pattern in HU shows that replication progresses bidirectionally around replication origins (Supplementary Figure S4C, top panels), and the Rtt101 pattern is similar (Supplementary Figure S4C, bottom panels).

Taken together, these results strongly suggest that Rtt101 colocalizes with replication forks, even in the absence of exogenous stress. Hence, it is tempting to hypothesize that a pool of Rtt101 constitutively travels with replisomes.

## Discussion

The repair of DNA–protein crosslinks has received great attention in the past decade, and proteolytic elimination of this damage has started to be well understood. Here, we provide evidence that the cullin E3 ubiquitin ligase Rtt101 mediates resistance to a variety of DPCs in the yeast *S. cerevisiae*. By monitoring the removal of an *in vivo* inducible DPC, we observed that Rtt101 facilitates DPC clearance from the DNA throughout the yeast cell cycle by ubiquitination event(s). We provide genetic evidence for a connection between Rtt101 and the protease Ddi1. Finally, we show that Rtt101 precisely follows the replisome on the DNA, even in unchallenged conditions.

### Parallel mechanisms for DPC ubiquitination

The findings presented in this study reveal a complex network of parallel pathways involved in the ubiquitination of DPCs. Notably, the absence of Rtt101 results in a significant reduction of ubiquitin in the proximity of Flp-cc (Figure 3F). A separate SUMO-dependent ubiquitination pathway orchestrated by the STUbL complex Slx5-Slx8 is also proficient in ubiquitinating the vicinity of Flp-cc (32). Similar to Rtt101, Slx5-Slx8 (hRNF4) complex promotes Top1ccs repair (80,81). Slx5-Slx8 STUbL generates mixed SUMO-Ub chains proposed to recruit Cdc48-Wss1 (32), thereby providing a rationale for the coexistence of Rtt101 within a pathway parallel to Wss1 (Supplementary Figures S1E and S2). This coexistence implies that Rtt101 might orchestrate the recruitment of repair factors independently of Wss1, as demonstrated by its functional interaction with Ddi1 (Figure 2E). The existence of two parallel ubiquitination pathways targeting Flp-cc is further supported by the observation that the loss of SUMO-dependent ubiquitination does not prevent the recruitment of Ddi1 to Flp-cc, confirming the presence of a distinct pathway that contributes to DPC repair by Ddi1 (32). Multiple ubiquitin-dependent pathways may serve specific roles, ideally targeting distinct sets of substrates, or potentially act in a compensatory manner to ensure the efficient repair of DPC lesions.

### The interplay of Rtt101 with Ddi1

Recently, the yeast aspartic protease Ddi1 has been associated with DPC repair, and current models envision that Ddi1 is targeted to DPCs when they become heavily modified with long ubiquitin chains (73), but it is unclear what ubiquitin ligase is responsible for Ddi1 targeting. Our data indicate that Rtt101 functions in the same pathway as Ddi1 in response to DPC drugs and that it is crucial for the chromatin enrichment of Ddi1 (Figure 2). We applied the ChEC-seq approach in order to investigate Ddi1 potential enrichment at inducible Flp-cc, similar to our analysis for Rtt101 (Figure 4). Unfortunately, this approach was technically unsuccessful, preventing us from concluding on the potential implication of Rtt101 in the recruitment of Ddi1 to Flp-cc (data not shown). While it is reasonable to speculate that Rtt101 may be the unidentified ubiquitin ligase involved in the recruitment of Ddi1 to DPCs, it is not possible to exclude that Rtt101 may also be important for the activity of Ddi1, as partially indicated by our genetic data. Ddi1 may also act in a Rtt101-independent manner, as loss of Rtt101 does not completely abrogate Ddi1 chromatin localization (Figure 2G). Given that Ddi1 is proposed to have a role during replication (24), we anticipated that the Rtt101 cullin might be restricted to S-phase to promote the action of Ddi1 on DPCs. Accordingly, Rtt101 travels on chromatin and follows the path of the replication fork (Figure 5B). We were therefore surprised to find that Rtt101 activity is not restricted to replicating cells, but rather seems to be important throughout the cell cycle (Figure 3D and E). This indicates that the Rtt101-Ddi1 axis could act on DPCs during or outside replication. Also, it would be interesting to define whether the activity of one of these pools holds greater significance.

### What are the targets of Rtt101-mediated ubiquitination in DPC repair?

Our findings indicate that the ubiquitin ligase Rtt101 is conferring resistance to various forms of DPCs, regardless of their association with DNA breaks (Figure 2). It also showed that Rtt101 is participating in the resistance to Top1ccs, and to camptothecin-induced Top1 degradation (Figure 1). Additionally, we provide evidence that ubiquitination in response to DPCs is, to some extent, dependent on Rtt101 (Figure 3F). Nonetheless, our study falls short in identifying the target(s) of Rtt101 ubiquitin ligase activity in the context of DPC repair. It remains challenging to anticipate whether Rtt101 modifies the adduct *per se*, or a factor standing near the crosslink site. Within the framework of Top1cc and Flp-cc, an obvious candidate is the crosslinked protein. This hypothesis is strengthened by the fact that CUL4B, the proposed functional homolog of Rtt101, is directly implicated in the ubiquitination of TOP1 after exposure to TOP1 poisons (40), mediated by the DCAF13 substrate receptor (75). We were unfortunately not able to demonstrate the direct ubiquitination activity of Rtt101 on Top1 following CPT-mediated trapping (Supplementary Figure S3B and S3C), yet we observe that Rtt101 influences Top1 stability and chromatin association (Figure 1G and H). While this result does not rule out the possibility of Rtt101 modifying Top1 and adducts, it may be attributed to suboptimal experimental conditions for elucidating such interactions. Our genetic data suggest that Top1 is not the source of toxicity when cells are exposed to non-enzymatic DPCs (Figure 1F and Supplementary Figure S1F). Therefore, Rtt101 likely possesses the ability to modify not only Top1 but also other types of DPC adducts. In higher organisms, the ubiquitin ligase TRAIP is associated with replisomes where it supports genome stability during replication (22,36,37,39). TRAIP promotes CMG bypass of DPCs by ubiquitination. In TRAIP-depleted cells, bypass of DPCs remains possible, albeit at a slower rate. The observations regarding the effects of Rtt101 reflect, in some aspects, the activity of TRAIP (Figures 3 and 5). Consequently, it remains plausible that Rtt101 modifies the adduct, similarly to mammalian TRAIP, and is necessary for ensuring the efficient bypass of DPCs during replication.

To date, the literature has described a limited number of substrates of Rtt101 ligase activity. Among these, Rtt101 ubiquitinates histone H3 within the acetylated H3-H4 heterodimer (82), a modification that ultimately controls histone deposition onto newly replicated DNA.

The significance of Rtt101-mediated ubiquitination of histones for chromatin structure and genome stability should not be underestimated. However, other aspects of Rtt101 function are probably relevant. In accordance with this view, analysis of mutated H3 in combination with *rtt101Δ* reveals a synergistic increase in sensitivity to DNA damage, including CPT (82), suggesting that the role of Rtt101 in response to Top1 poisoning extends beyond ubiquitination of histones. This notion can likely be extended to other types of DPCs.

### Regulation of Rtt101 by subassembly formation

Our data indicate that Rtt101 is closely associated with replisomes (Figure 5). Given the variety of adducts potentially blocking replisomes, Rtt101 should consequently possess a broad specificity, enabling it to target various crosslinked proteins. Indeed, Rtt101 provides resistance towards different DPC types, which differ in chemical structure, DNA context, etc. (Figures 1, 2 and 3). This implies that Rtt101 is tightly regulated, not only to avoid unwanted ubiquitination but also for effective targeting of DPCs.

One way to achieve such broad specificity would be the numerous adaptors capable of forming subassemblies with Rtt101 (42,43). Given that the subassemblies can include at least five different substrate adaptors (Figure 1C; Esc2, Ctf4, Orc5, Crt10 and Rtt107), bridged by the linker protein Mms1 and occasionally Mms22 (42,43), there are numerous possible arrangements. Notably, these components of Rtt101 subcomplexes are genetically relevant after inactivation of other repair pathways (Wss1 and Tdp1), supporting this idea ((24), Figure 1B). Future investigations are required to understand which Rtt101 subcomplexes are required to recognize the diverse targets in the framework of DPC repair.

We reasoned that a fraction of Rtt101 is traveling with replisomes (Figure 5), but whether it does so in an active form or in a pre-assembled form was not investigated. Considering the requirement to recognize a wide range of DPCs potentially obstructing forks, it is conceivable that several forms of active Rtt101-based ligases accompany replication. One could envision that a pre-assembled Rtt101^Mms1^ complex associates with replisomes, and relevant adaptors are recruited subsequently depending on the nature of the DPC encountered. Indeed, our data confirm the essentiality of the interaction between Rtt101 and Mms1 for DPC resistance (Figure 2B). Also, experiments in yeast showed that Mms22 interacts at the same time with replisome components and Rtt101, even in unchallenged conditions, an interaction potentially channeled by Ctf4 (45). Another option is that Rtt101 is recruited *de novo* and is not constitutively associated with replisomes. In support of this last hypothesis, Rtt101 is also active outside of replication (Figure 3E), where replication cannot sense DPCs. Finally, it may be that only a fraction of Rtt101 associates with replisomes, or that Rtt101 only associates with a fraction of replisomes.

### A model for Rtt101 action during DPC repair

**Figure 6.**
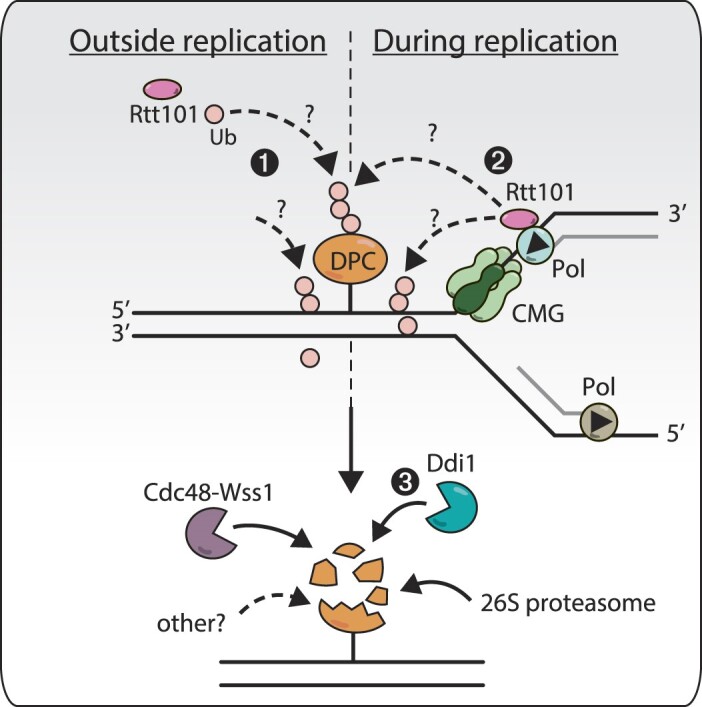
Hypothetical model of the action of Rtt101 across the yeast cell cycle. Rtt101 promotes the removal of DNA–protein crosslinks (DPCs) from the DNA throughout the cell cycle in yeast. In the absence of DNA replication (left panel), Rtt101 ubiquitinates an unknown target around DPCs (1). During DNA replication (right panel), a pool of Rtt101 travels with replisomes and will similarly ubiquitinate the vicinity of DPC barriers upon collision with replication forks, by modifying the adduct or another factor nearby (2). Whether outside or during DNA replication, Rtt101-mediated ubiquitination recruits the ubiquitin-dependent protease Ddi1 (3), facilitating DPC repair. Other DPC repair mechanisms are also ongoing in parallel, notably the Cdc48-Wss1 axis, the 26S proteasome, and potentially other currently unknown mechanisms.

Our data show that the ubiquitin ligase Rtt101 takes part in the repair of DPCs; it is required at different stages of the budding yeast cell cycle, and it genetically closely interacts with the ubiquitin-dependent protease Ddi1. We suggest a model (Figure 6) in which Rtt101^Mms1^ subcomplex travels with replisomes and ubiquitinates DPC barriers upon collision. It remains unclear whether Rtt101 ubiquitinates the DPC moiety or a factor at the lesion site. The activity of Rtt101 will allow replication to resume through several potential mechanisms. It may promote damage bypass, replisome uncoupling or adduct degradation by promoting recruitment of factors such as Ddi1. This hypothetical role of Rtt101 resembles its proposed function in replication restart or repriming events, enabling lesion bypass during S-phase (45). To do so, Rtt101 mediates Mrc1 ubiquitination, promoting repair through HR-mediated mechanisms. A similar process might be at play when Rtt101 encounters DPCs, potentially allowing their bypass. In the absence of Rtt101, a slower alternative mechanism ultimately facilitates replication restart. Outside of replication, Rtt101 also ubiquitinates persistent DPCs *via* its replication-independent pool. This underscores the broad significance of Rtt101 throughout the cell cycle, by promoting the removal of DPCs and contributing to the maintenance of genomic integrity.

## Supplementary Material

gkae658_Supplemental_Files

## Data Availability

The accession number for the data reported in this study is GEO: GSE247492. Raw fastq files and processed bigwig files have been uploaded as well as the sacCer3-Flp-nick fasta file and the early and late ACSs coordinates. Further information and requests for resources and reagents should be directed to and will be fulfilled by Françoise Stutz: francoise.stutz@unige.ch.
