# Supplementary material for: The cullin Rtt101 promotes ubiquitin-dependent DNA−protein crosslink repair across the cell cycle
Source: Nucleic Acids Res. 2024 Jul 30;52(16):9654–70. doi: 10.1093/nar/gkae658 (PMC11381328; doi:10.1093/nar/gkae658)
Supplement: gkae658_Supplemental_Files [file gkae658_supplemental_files.zip › NAR-03660-J-2023_Supplementary Figures and Legends.pdf]

Supplementary Figure 1

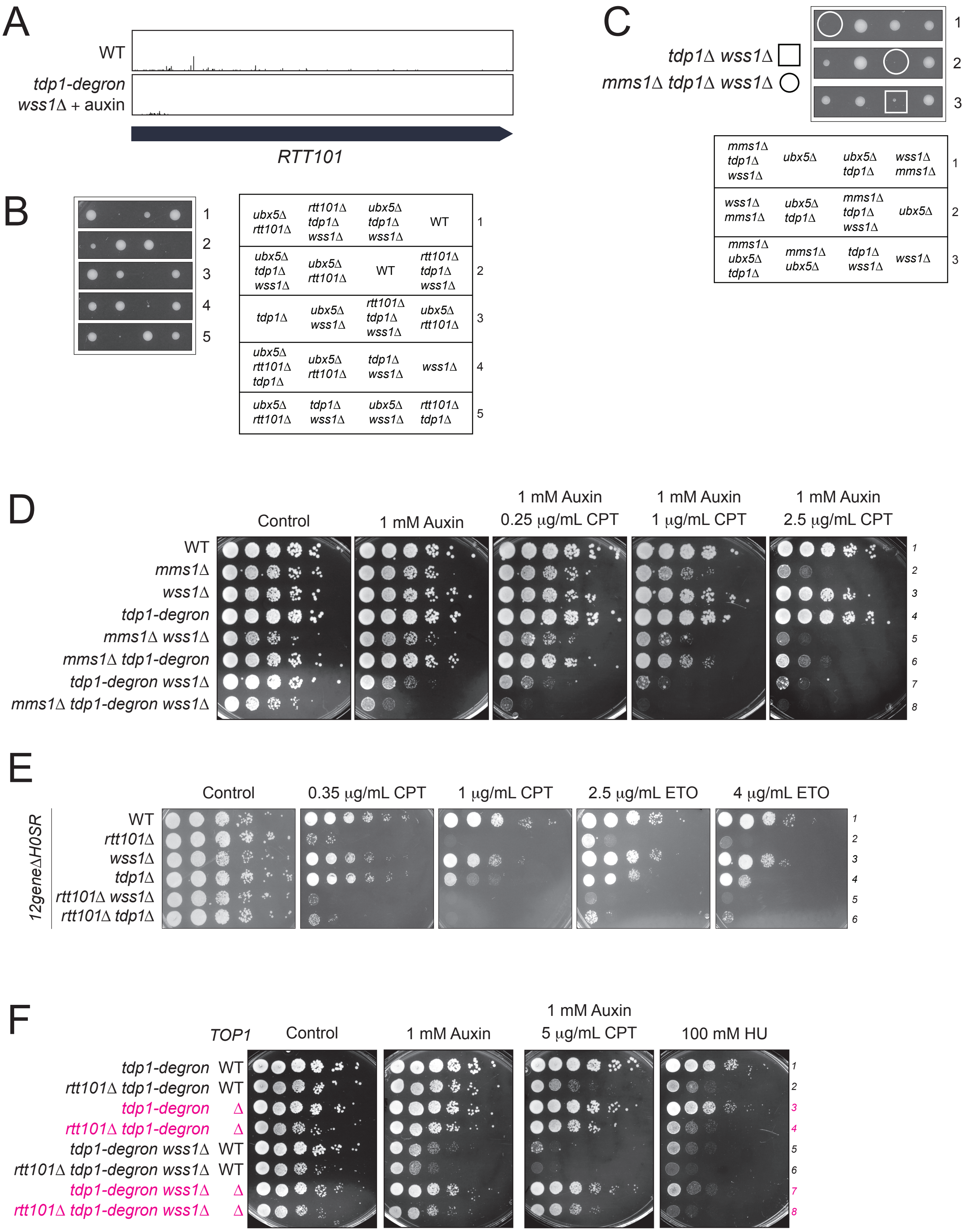

Supplementary 1 (related to Figure 1)

(A) Snapshot depicting transposon coverage over the *RTT101* gene body in the *tdp1-degron wss1*Δ + auxin and one of the WT libraries. The height of the bars represents the number of reads for each transposon.

(B) Full genotyping of yeast tetrads presented in Figure 1E. Tetrads were analyzed after dissection of the diploid *[TDP1/tdp1Δ; WSS1/wss1Δ; UBX5/ubx5Δ; RTT101/rtt101Δ].*

(C) Loss of *MMS1* affects growth of *tdp1Δ wss1Δ*. Tetrads were analyzed after dissection of the diploid *[TDP1/tdp1Δ; WSS1/wss1Δ; MMS1/mms1].*

(D) Transposon screen validation of *MMS1*. Cells were grown in YEPD and spotted on a medium supplemented with 1 mM auxin and various concentrations of Camptothecin (CPT). Plates were incubated for 2 days at 30°C.

(E) Rtt101 and Wss1 are both required for resistance against enzymatic Top1- and Top2-DPCs. The *12geneΔ0HSR* mutant (1) was used to reveal sensitivities to trapped Top1 (CPT) and Top2 (ETO). Cells were grown in YEPD and spotted on a medium supplemented with indicated concentrations of CPT and ETO. Plates were incubated for 2 days at 30°C.

(F) Top1 crosslinks are not the only cause of cell sensitivity to genotoxins in the absence of Rtt101. *TOP1* deletion specifically rescues CPT but not HU sensitivity of *tdp1-degron wss1Δ* and *rtt101Δ tdp1-degron wss1Δ* mutants. Cells were grown in liquid YEPD and plated on 1 mM auxin for *tdp1-degron* depletion, 5 µg/mL CPT, 100 mM HU. Plates were incubated for 2 days at 30°C.

Supplementary Figure 2

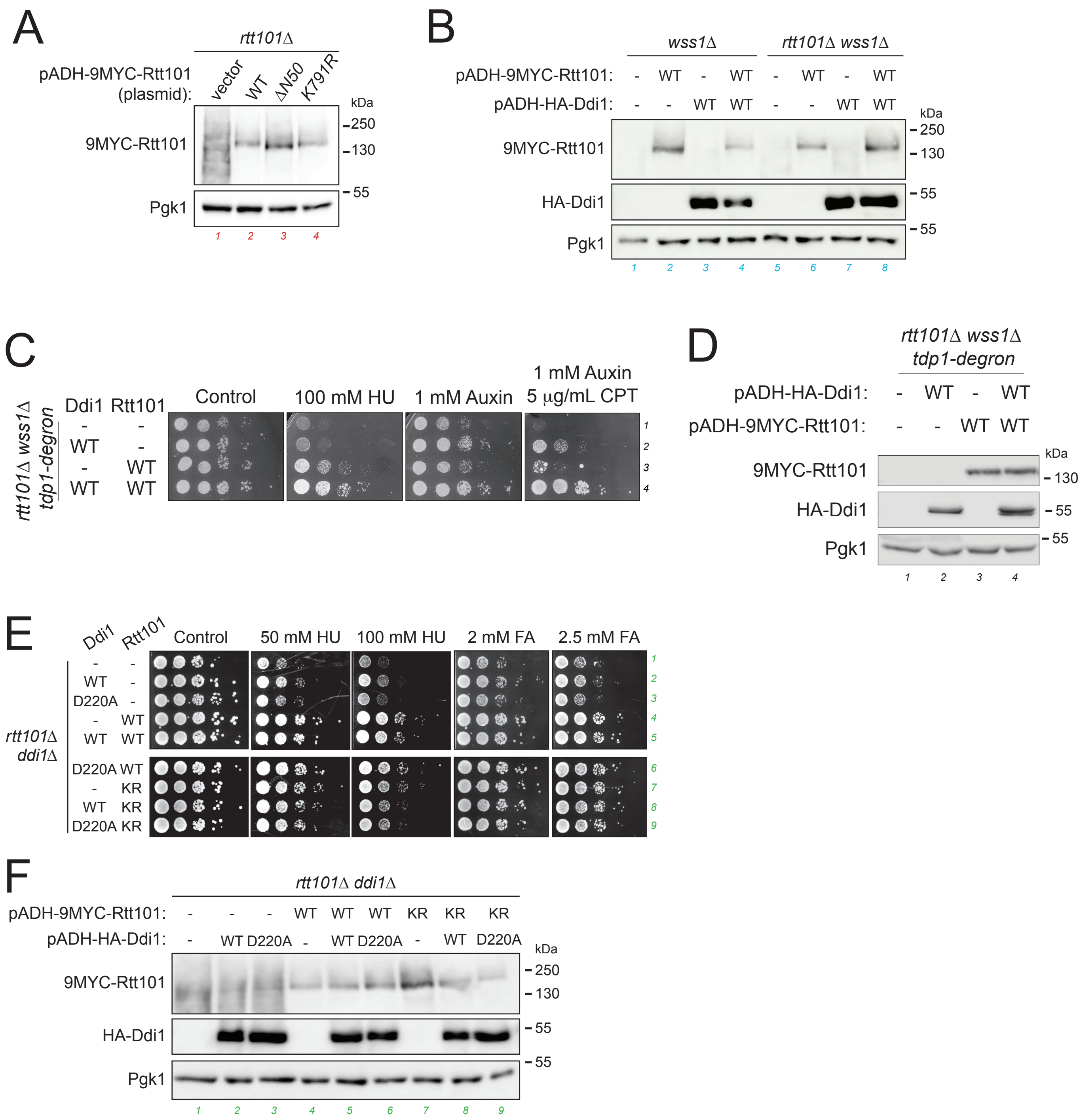

Supplementary 2 (related to Figure 2)

(A) Western blot protein levels of 9MYC-Rtt101 variants expressed from plasmids under the strong pADH promoter (used in Figure 2B). Pgk1 was used as a loading control. Signals were revealed by chemiluminescence.

(B) Protein levels of HA-Ddi1 and 9MYC-Rtt101 (associated with Figure 2E). Pgk1 was used as a loading control. Signals were revealed by chemiluminescence.

(C) Ddi1 is less proficient in alleviating the *tdp1wss1* phenotype on HU and CPT in the absence of the cullin Rtt101. Mutant *rtt101Δ tdp1-degron wss1Δ* was co-transformed with plasmids overexpressing HA-Ddi1, 9MYC-Rtt101, or both. See S2D for western blot analysis of protein levels.

(D) Western blot showing the protein levels of HA-Ddi1 and 9MYC-Rtt101, performed similarly to S2A.

(E) Genetic analyses of *ddi1-D220A* and *rtt101-K791R* mutants on HU and FA. The *rtt101Δ ddi1Δ* mutant was co-transformed with plasmids overexpressing HA-Ddi1, 9MYC-Rt101, or both. All the mutants were spotted on the same plate. See S2F for western blot analysis of associated protein levels.

(F) Western blot showing the protein levels, similar to S2A.

Supplementary Figure 3

A

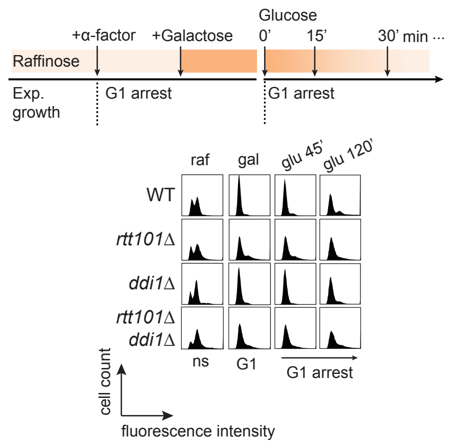

B

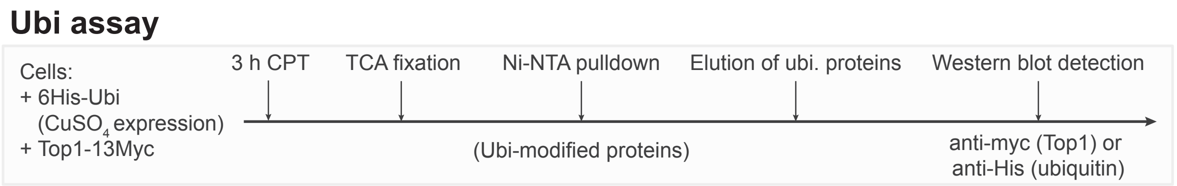

C

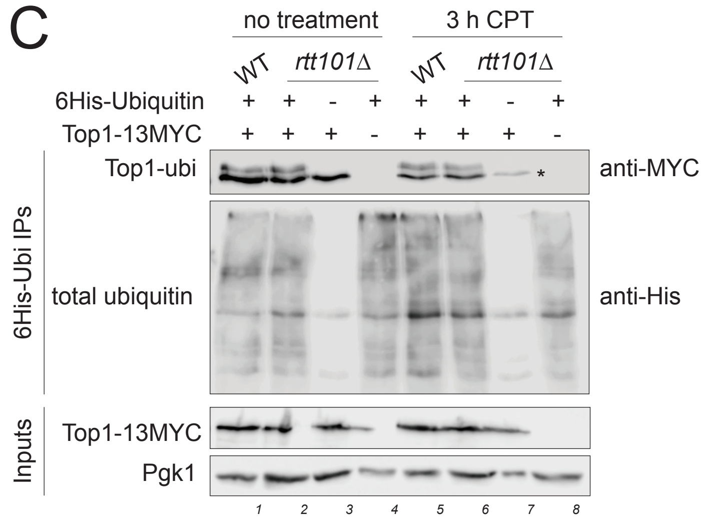

Supplementary 3 (related to Figure 3)

(A) FACS analysis of cells kept in G1 after glucose release and repression of Flp-cc expression. Experimental design applied in (Figure 3E) for measuring Flp-cc removal in G1 phase of the cell cycle. The experiment was performed as in (Figure 3D), except that after Flp-cc induction with galactose in G1-arrested cells, cells were transferred in glucose-containing medium with α-factor to keep them in G1. FACS profiles at representative time points are shown in the bottom panel.

(B) Workflow representation of the Ubiquitin assay used to detect Top1-ubiquitination. Cells were pre-grown in medium containing $CuSO_4$ to induce His_6-Ubiquitin expression from transformed plasmid. Cells were TCA fixed before lysate preparation. Ubiquitin-associated species were recovered by Ni-NTA agarose beads pulldown. Top1 is tagged at the genomic locus with 13MYC. The experiment was performed in the *12geneΔ0HSR*. Cells were treated for 3 h with 1.5 µg/mL CPT.

(C) Ubiquitination status of Top1 is neither affected by the loss of the ubiquitin ligase Rtt101 nor by CPT treatment. Ubiquitinated levels of Top1 evaluated by the Ubi assay.

Supplementary Figure 4

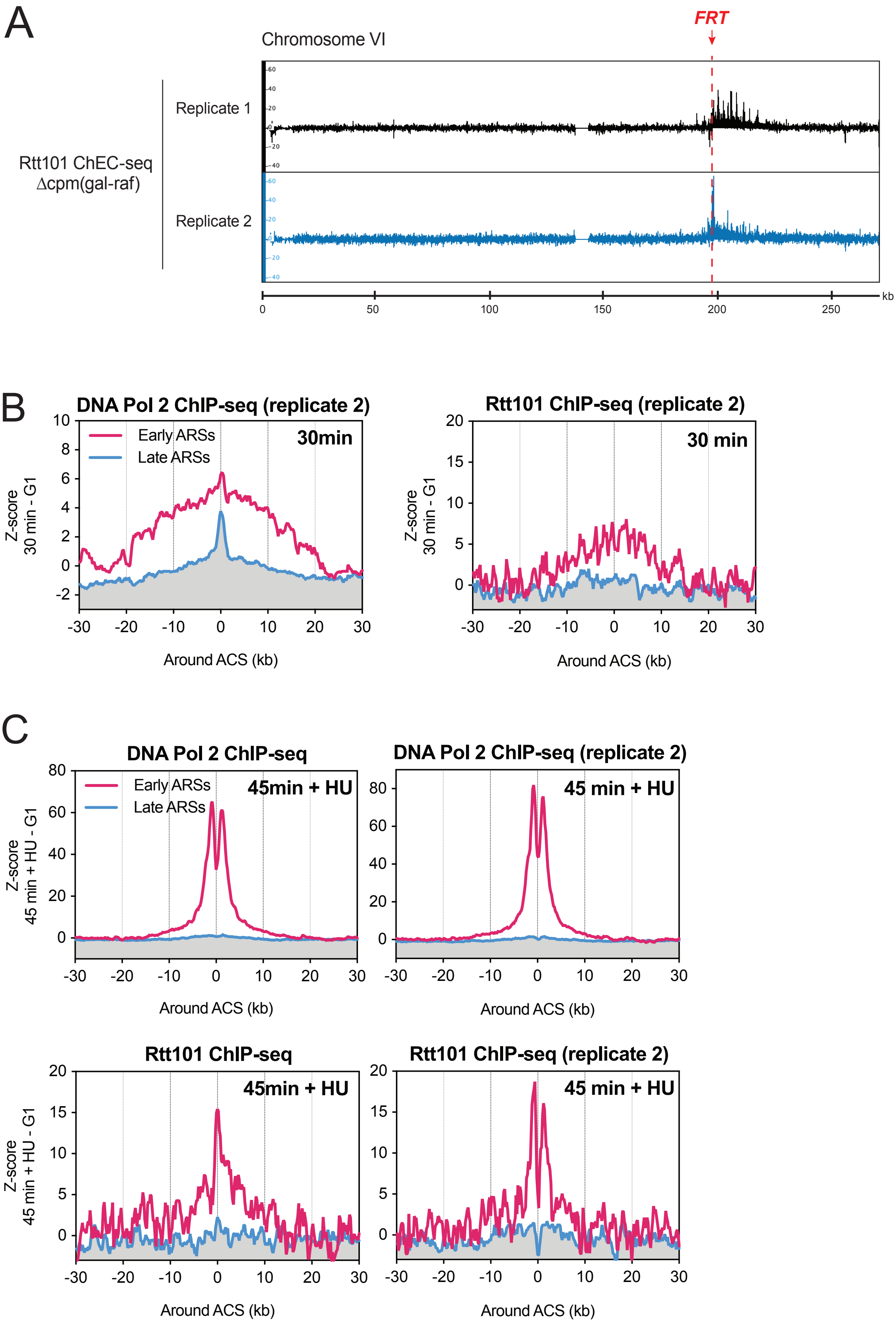

Supplementary 4 (related to Figures 4 and 5)

(A) Tracks of Rtt101 signals on chromosome VI by ChEC-seq in two independent replicates (mean is presented in Figure 4D). Data are presented as (Galactose – Raffinose) difference in cpm. Location of the *FRT* site is indicated by the red arrow.
(B) Metagene plots depicting the ratio of DNA Pol 2 and Rtt101 binding to chromatin in S-phase (30 min) over their respective binding in G1-phase (second replicate). Plots were centered on the oriented ACS taking separately early ARSs (n = 40) and late ARSs (n = 226).
(C) Metagene plots depicting the ratio of DNA Pol 2 and Rtt101 binding to chromatin in S-phase in challenged conditions (45 min + HU) over their respective binding in G1-phase (2 replicates). Plots were centered on the oriented ACS taking separately early ARSs (n = 40) and late ARSs (n = 226).

References

1. Chinen, T., Ota, Y., Nagumo, Y., Masumoto, H. and Usui, T. (2011) Construction of multidrug-sensitive yeast with high sporulation efficiency. *Biosci Biotechnol Biochem*, **75**, 1588-1593.
